# A214 WOMEN WITH INFLAMMATORY BOWEL DISEASE HAVE INCREASED HEALTH-CARE UTILIZATION DURING PREGNANCY AND POSTPARTUM COMPARED TO THOSE WITHOUT INFLAMMATORY BOWEL DISEASE: A POPULATION-BASED COHORT STUDY

**DOI:** 10.1093/jcag/gwac036.214

**Published:** 2023-03-07

**Authors:** P Tandon, V Huang, D Feig, R Sakin, C Maxwell, Y Gao, D Fell, C Seow, J Snelgrove, G C Nguyen

**Affiliations:** 1 University of Toronto; 2 ICES, Toronto; 3 CHEO, Ottawa; 4 University of Calgary, Calgary, Canada

## Abstract

**Background:**

Compared to those without inflammatory bowel disease (IBD), women with IBD may have increased health-care utilization during pregnancy and postpartum. This may lead to significant morbidity and decrease in quality of life. Characterizing this health-care use is important for health-policy purposes to determine methods to shift care to the ambulatory setting.

**Purpose:**

We aimed to compare health-care utilization of women with and without IBD during preconception, pregnancy and postpartum.

**Method:**

We accessed administrative databases and validated algorithms at the Institute of Clinical Evaluative Services (ICES) in Ontario to identify women (age 18-55) with and without IBD who had a completed live, singleton pregnancy between 2003 and 2018. The primary outcome was to characterize differences in emergency department (ED) visits and hospitalizations between women with and without IBD during the 12 months preconception, pregnancy, and in the 12 months postpartum. The secondary outcome was to assess differences in prenatal care between women with and without IBD. Multivariable negative binomial regression with generalizing estimating equations, accounting for multiple pregnancies for each patient, was performed to report incidence rate ratios (IRR) with 95% confidence intervals (95% CI). Covariates included maternal age at conception, location of residence at conception (rural vs. urban), socioeconomic status (using surrogate marker of neighborhood income quintile), and maternal comorbidity.

**Result(s):**

9158 pregnancies in 6163 women with IBD and 1,729,411 pregnancies in 1,091,013 women without IBD were included. Women with IBD were older at time of delivery and had greater pre-pregnancy comorbidities. During pregnancy, women with IBD were more likely to visit the ED (IRR 1.13, 95% CI,1.08-1.18) and be hospitalized (IRR 1.11, 95% CI,1.01-1.21) for non-IBD specific reasons. Similarly, during postpartum, women with IBD were more likely to visit the ED (IRR 1.21, 95% CI, 1.15-1.27) and be hospitalized (IRR 1.18, 95% CI, 1.05-1.32) for non-IBD specific reasons. Venous thromboembolic events accounted for 7.0% of all postpartum hospitalizations in women with IBD compared to 2.7% in those without IBD (p<0.0001). There was no difference in ED visits and hospitalizations between women with and without IBD in preconception. Finally, women with IBD had greater number of prenatal visits with obstetricians during pregnancy and were more likely to receive a first trimester prenatal visit compared to those without IBD.

**Conclusion(s):**

Compared to those without IBD, women with IBD are more likely to visit the ED and be hospitalized during pregnancy and postpartum, particularly for venous thromboembolic events. Efforts should be made from a health policy perspective to increase access to ambulatory care for patients with IBD during the peripartum period which in turn may reduce acute setting health-services utilization.

**Please acknowledge all funding agencies by checking the applicable boxes below:**

CCC

**Disclosure of Interest:**

None Declared

